# Longitudinal sampling of the lung microbiota in individuals with cystic fibrosis

**DOI:** 10.1371/journal.pone.0172811

**Published:** 2017-03-02

**Authors:** Fiona J. Whelan, Alya A. Heirali, Laura Rossi, Harvey R. Rabin, Michael D. Parkins, Michael G. Surette

**Affiliations:** 1 Department of Biochemistry and Biomedical Sciences, McMaster University, Hamilton, Canada; 2 Department of Microbiology, Immunology and Infectious Diseases, The University of Calgary, Calgary, Canada; 3 Department of Medicine, The University of Calgary, Calgary, Canada; 4 Department of Medicine, McMaster University, Hamilton, Canada; Lee Kong Chian School of Medicine, SINGAPORE

## Abstract

Cystic fibrosis (CF) manifests in the lungs resulting in chronic microbial infection. Most morbidity and mortality in CF is due to cycles of pulmonary exacerbations—episodes of acute inflammation in response to the lung microbiome—which are difficult to prevent and treat because their cause is not well understood. We hypothesized that longitudinal analyses of the bacterial component of the CF lung microbiome may elucidate causative agents within this community for pulmonary exacerbations. In this study, 6 participants were sampled thrice-weekly for up to one year. During sampling, sputum, and data (antibiotic usage, spirometry, and symptom scores) were collected. Time points were categorized based on relation to exacerbation as Stable, Intermediate, and Treatment. Retrospectively, a subset of were interrogated via 16S rRNA gene sequencing. When samples were examined categorically, a significant difference between the lung microbiota in Stable, Intermediate, and Treatment samples was observed in a subset of participants. However, when samples were examined longitudinally, no correlations between microbial composition and collected data (antibiotic usage, spirometry, and symptom scores) were observed upon exacerbation onset. In this study, we identified no universal indicator within the lung microbiome of exacerbation onset but instead showed that changes to the CF lung microbiome occur outside of acute pulmonary episodes and are patient-specific.

## Introduction

Cystic fibrosis (CF) is caused by mutations in the cystic fibrosis transmembrane conductance regulator (CFTR) gene [1]; [2], which leads to impairments in pancreatic and liver function, and intestinal obstruction [3]; [4]. However, it is the effect that this disease has on the lungs that has the greatest clinical burden. Repeated cycles of airway infection, mucous impaction, and bronchiectasis results in the majority of morbidity and mortality in the patient population [3]; [5]. This chronic lung disease is progressive, manifesting as persistent lung function decline and diminishing quality of life [6]; [7].

Pulmonary exacerbations are respiratory perturbations characterized by increased respiratory symptomatology, systemic inflammation, fatigue, and weight loss [8], symptoms which are potentially rescued by airway clearance and antimicrobial therapy directed against chronically infecting pathogens [5]; [9]; [10]; [11]. These events are critical in CF as they cause permanent loss of lung function; however, the mechanisms underlying these events remain largely unknown. Exacerbations have been associated with viral infections [12]; [13] as well as changes in density of primary bacterial pathogens within the lungs [12]; [14] perhaps due to a clonal expansion of pre-existing strains [15]. However, the true cause of pulmonary exacerbations is likely multi-factorial in nature, including interactions between the immune system, lung microbiota, airway physiology, and the environment [5]; [12], complicating the understanding and treatment of these events.

The CF airways have long been known to harbor certain primary pathogens such as *Pseudomonas aeruginosa*, *Burkholderia cepacia* complex, and *Staphylococcus aureus* [16]. More recently, as sequencing technologies and laboratory culture techniques advance, it has become appreciated that there are many additional bacterial members of the CF lung community which have the propensity to contribute to disease. For example, *Stentrophomonas maltophilia*, *Achromobacter* spp., *Mycobacterium abscessus*, Methicillin-resistant *Staphyloccocus aureus* (MRSA), and the *Streptococcus* Anginosus/Milleri group have been described as emerging CF pathogens [16]; [17] [18]; [19]; [20]. Similarly, other non-bacterial members of the CF lung microbiome have been implicated in worsened prognosis such as the fungus *Aspergillus fumigatus* [16]; [20].

To date, many studies of the CF lung microbial population, or microbiome, include comparisons of sputum samples collected during pulmonary exacerbation and clinical stability (for e.g. [21]; [14]). While these sampling methods can be very informative, they cannot determine daily dynamics of the CF lung microbiome during exacerbation onset. There are two notable exceptions; Carmody *et al*. collected daily sputum samples from 4 participants over a 25-day period which included the onset of pulmonary exacerbation [22]. In this study, the authors identified changes in the CF microbiome at exacerbation onset in a subset of participants by examining the beta diversity dissimilarity between longitudinal bacterial communities [22]. Second, Cuthbertson *et al*. studied 10 CF patients at baseline, 30 days prior to treatment, treatment for exacerbation, 30 days post treatment, and post-exacerbation baseline [23]. The authors determined that the core microbiota were resistant to exacerbation and associated antimicrobial treatments [23].

In this study, we expand on the above by examining relative changes to the CF lung bacterial community over the course of one year in 6 participants in the context of clinical status (exacerbation treatment versus stability), changes in participant reported symptom scores and spirometry values, and antibiotic treatments. We discovered no consistent indicator of exacerbation onset and instead confirm the patient-specific nature of the CF lung microbiome.

## Materials and methods

### Participant recruitment and sputum collection

From July to October of 2012, 6 knowledgeable and compliant cystic fibrosis (CF) patients were recruited for this study from the Southern Alberta Adult Cystic Fibrosis Clinic. The median age of participants was 32.5 (IQR 26–36), and all were homozygous for the F508del mutation except for one who was a compound heterozygote, F508del/621+1G-T (Table 1). Median lung function for participants was 1.72L (IQR 1.55L-2.66L), 66.9% predicted (IQR 58.3–85.0). Participants self-collected sputum samples 3 times a week (Monday, Wednesday, and Friday) into clinical laboratory collection jars (which were then immediately stored in their home freezers). During periods of absence from home (e.g. holidays/work trips) some samples were omitted. Participants self-reported data including symptom scores adapted from Jarad *et al*. [24]. Symptoms with respect to Cough, Sputum Production, Shortness of Breath, Wheezing, Nasal Irritation, Throat Irritation, Fatigue, and Appetite were independently scored relative to an individual's norm/baseline (= 0) with increased symtomatology scored as 1 = mild, 2 = moderate, or 3 = severe deterioration. Additionally, study participants were outfitted with PIKO-6 (nSpire Health; Longmont CO) home spirometers to measure spirometry. Prior to enrolment, all participants were trained by a study investigator in the use of the PIKO-6 device. Participants were taught to perform expiratory manauvers three times and record each value. Values used represent the best of each three attempts. Values collected at enrolment were correlated with complete pulmonary function testing performed during the clinic visit. Lung function values were reported as forced expiratory volume in one (FEV1) second. Any antibiotics, including those for chronic suppression of lung disease and acute management of pulmonary exacerbation, were similarly recorded. All collected data and records of antibiotic usage were made available to the study authors. Ethical approval for this study was given by the Calgary Health Region Ethics Board (REB-24123). At the enrolment visit, each patient provided written informed consent (with an REB approved document) after detailed discussion with research/clinic staff regarding what the study entailed. Of the 6 participants who began the study, 3 completed the full 1-year term with the remaining 3 participants dropping out of the study due to poor health or non-compliance (S1 Table); however, all 6 contributed serial samples and are included in subsequent analyses.

**Table 1:** Clinical and methodological information about the study participants.

Participant	Age at Study Onset	CFTR Mutation	Clinically cultured & treated primary CF pathogen(s)	% predicted lung function (FEV1/FVC) at study onset	# of Exacerbations
A*	36	F508del/F508del	P.*aeruginosa*, M.*abscessus*	54.0	1
B	38	F508del/F508del	P.*aeruginosa*	73.5	1
C	26	F508del/F508del	*P*.*aeruginosa*, *S*. *agalactiae*	105.0	0
D*	36	F508del/F508del	P.*aeruginosa*, *Streptococus* Anginosus group	58.3	1
E	23	F508del/621+1G->T	P.*aeruginosa*	60.2	4
F*	29	F508del/F508del	P.*aeruginosa*, S.*aureus*, *Cupriavidus*.*sp*	85.0	1

* = did not complete study

At the end of the study, participants returned a study log which included the metadata and their stored sputum using -20°C freezer packs and insulated transport bags to ensure samples were kept frozen. Upon receipt, samples were immediately transferred and stored at -80°C.

Samples were assigned into one of the three categories based on the time of collection: *Treatment* if the sample was collected during a pulmonary exacerbation (as defined by Fuchs *et al*. [8]) and after any intravenous antibiotics were administered; *Intermediate* if the sample was collected in the month prior to or following a pulmonary exacerbation; *Stable* otherwise. At the end of the study, we retrospectively chose a subset of samples for marker gene analysis. Where possible, we chose a subset that included tri-weekly samples from the Treatment stage, weekly samples during Intermediate stages, and monthly samples during Stable periods. Using this schema, 121 of the 508 available samples were chosen for 16S rRNA gene sequencing (S1 Table).

### Clinical microbiology

Standard clinical microbiology was performed during regular clinic visits as has been previously described [18] [25]. Quantitative analysis of sputum was carried out by plating on Columbia blood agar (CBA), chocolate agar (CHOC), MacConkey agar (MAC), mannitol-salt agar (MSA), and oxidation-fermentation polymyxin bacitracin lactose agar (OFPBL). These solid media plates were incubated at 35°C, 5% CO_2_ for 2 days with the following exceptions: OFPBL was incubated at 30°C; CHOC which was incubated anaerobically.

### DNA isolation and illumina sequencing

Genomic DNA was isolated as previously described [26]; [27]. Methods of genomic DNA extraction differed from [26] only in that the starting material was 300μl of sputum which had been homogenized by repeated passage through a 18 gauge needle and syringe. Barcoded universal primers adapted from [27] were used to amplify the variable 3 region of the 16S rRNA gene. The PCR reaction consisted of 5pmol of each primer, 50ng template DNA, 200μM dNTPs, 1.5mM MgCl_2_, 4mg/mL BSA, 1x reaction buffer, and 1 U Taq polymerase. The PCR protocol was as follows: 94°C for 5 minutes, followed by 30 cycles of 94°C for 30 seconds, 50°C for 30 seconds, and 72°C for 30 seconds, with a final 72°C for 7 minutes. Presence of a PCR product was verified by electrophoresis (2% agarose gel). PCR products were normalized for quantity using the SequalPrep Normalization kit (ThermoFisher #A10510-01) and sequenced using the Illumina MiSeq platform using 2x250 paired-end reads.

### 16S rRNA sequence processing and analysis

The resulting sequencing data were processed using a custom in-house pipeline as in [26] with some modifications (S1 Fig). Briefly, primers and/or read-through of the variable 3 region was trimmed using cutadapt [28], low-quality reads were culled using sickle with a quality threshold of 30 (https://github.com/najoshi/sickle), and chimeras were removed using USEARCH as written into QIIME [29]; [30]. Operational taxonomic units (OTUs) were generated using AbundantOTU+ [31] and each was given a taxonomic assignment using the RDP Classifier [32] against the Greengenes reference database (February 4th 2011 release) [33]. OTU tables were generated via QIIME [30]. Any OTU consisting of only one read across the dataset (i.e. singleton) was removed. After processing, there was a mean of 105,884 reads per sample (range: 33,940–215,072) and a mean of 216 OTUs per sample (range: 73–491). The 16S rRNA gene sequencing data and clinical metadata that makeup this dataset are available via NCBI’s Short Read Archive, (BioProject PRJNA360332).

All analyses of the resulting OTU table were performed in R (R Core Team 2016) using packages phyloseq [34] and vegan for beta diversity calculations, vegan for tests of community-wide significance (i.e. PERMANOVA), and pheatmap to generate heatmap figures. A UPGMA representation of the Bray-Curtis dissimilarity between samples was generated using QIIME. Phylogenetic representations of participant's OTU diversity were generated by trimming the reference phylogeny provided with the 2011 release of Greengenes to those taxa which matched taxonomic assignments of each participant's OTUs. Visual representations of these phylogenies were created using graphlan [35]. Correlations of core OTUs, as defined by any OTU present in all samples from a particular participant with a sum of >1.0% over the study period, and key collected data (symptom scores, FEV1, antibiotic usage, alpha, and beta diversity) were calculated using eLSA [36] and were considered significant if p-values < 0.05 and q-values < 0.05, and the length of the observed correlation spanned the full dataset.

## Results

### Participant information and samples collected

In total, 6 individuals took part in this study. All 6 participants were chronically colonized with *Pseudomonas aeruginosa*; additionally, 4 of the 6 participants were chronically infected with additional organisms being targeted with antibiotic therapy: *Mycobacterium abscessus*, *Streptococcus agalactiae*, *Staphylococcus aureus*, *Cupriavidus sp*., or *Streptococcus* Anginosus/Milleri group (Table 1). *Streptococcus* Anginosus/Milleri group members have been previously reported as common CF pathogens in this clinic [17]. These individuals experienced a total of 8 exacerbations (Table 1).

**Fig 1:**
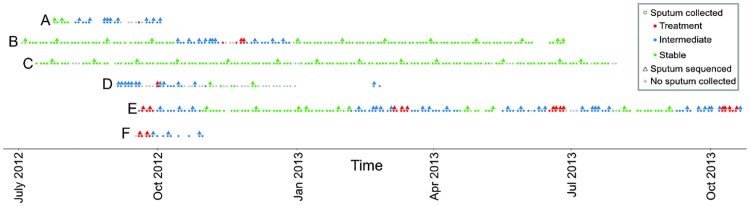
Outline of sputum collection and samples chosen for sequencing. Participants self-collected sputum 3 times a week while simultaneously recording clinical symptoms. On occasion, sputum could not or was not collected yet participant information was recorded (gray dots). Samples were retroactively chosen for microbiome analysis based on the sample type, aiming to follow Treatment more closely (1 sample/per 2–3 days) then Intermediate (1 sample per 1 week) and Stable (1 sample per 1 month) samples. All but one participant, C, experienced an exacerbation during the study period. Exact dates and length of sample collection for each participant is provided in S1 Table.

Of the 6 participants who began the study, 3 completed the year-long term with an average of 150 samples over the study period (Fig 1, S1 Table). The remaining participants dropped out of the study after an average of 116 days, and 20 samples per patient (S1 Table, Fig 1). A subset of this collection were retrospectively chosen for 16S rRNA gene sequencing with a focus on tri-weekly Treatment samples, weekly Intermediate samples, and monthly Stable samples (Fig 1).

### The CF lung microbiome is patient-specific

**Fig 2:**
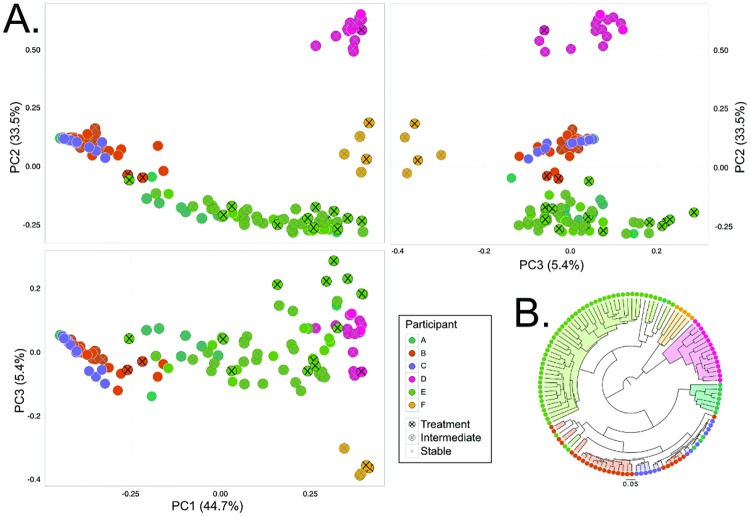
The CF lung microbiome is distinguished by individual. **A**. PCoA plots of all participants illustrate the clustering of participant samples, indicated as significant by PERMANOVA (p = 0.001). Health state within participants, as defined as Stable, Intermediate (<1 month pre- or post-Treatment), and Treatment was significant (PERMANOVA, p = 0.016), but was highly confounded by the participant (p = 0.042 of Participant:Health interaction term). **B**. UPGMA phylogeny depicting the Bray-Curtis dissimilarity between samples. It is apparent that the principle driver of similarity between samples are inter-individual microbial lung composition due to the almost complete separation of participant samples. PC = Principal Coordinate.

First, we aimed to examine the study-wide diversity amongst samples at the community level. Using the Bray-Curtis dissimilarity metric, which takes the relative abundance of individual OTUs into account, it was shown that the lung microbiota was significantly different between participants (PERMANOVA, p = 0.001). This result is visualized using a Principal Coordinates Analysis (Fig 2a). In a few cases, such as between Participant B and C, these participant-specific clusters overlap, indicating shared elements of their microbial composition. This is further examined via a genus-biplot of the PcoA (S2 Fig) which indicates that *Pseudomonas* contributes to the separation of samples from Participants B and C; similarly, *Staphylococcus* and *Cupriavidus* separates samples from Participant F, and *Fusobacterium* separates samples from Participant D. Further, a UPGMA phylogeny of the Bray-Curtis dissimilarity between samples shows almost perfect inter-participant separation (Fig 2b).

Additionally, we investigated the effect of sample type (Treatment, Intermediate, Stable) on the microbiota at the community level by PERMANOVA (p = 0.016). Although sample type was found to have a significant effect on microbial composition, this result was confounded by the participant (p = 0.042 of the Participant:Health interaction term). This indicates that the composition of the microbiome is influenced more by the individual then by the sample type as has been previously shown (for e.g. [21], [37]).

### Exacerbation does not consistently associate with community-wide changes to the microbiome

**Fig 3:**
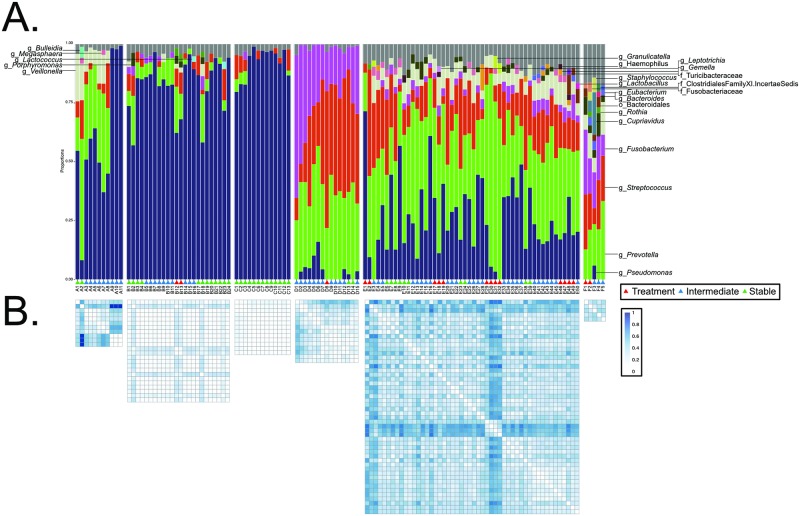
The effects of exacerbation on the lung microbiome are not consistently seen at the community level. **A**. Taxonomic summaries of all samples sequenced. These summaries indicate that changes to the lung microbiome upon exacerbation are not often obvious when examining the community-wide taxa composition. Taxa present at <2% are summarized in the gray bar. Participant E experienced 4 exacerbations during the study period which are indicated with black lines. **B**. Heatmaps indicate the Bray-Curtis dissimilarity between each sample. Here, we can see that samples taken during some exacerbations are more dissimilar to those collected during stability; however, this is not true for every exacerbation. These observations are qualified by statistical measures (S2 Table) and were independent of FEV1 (S3 Table).

Next, we examined each participant's microbiota independently. Taxonomic summaries were used to visualize changes in the microbiota over the course of the study and in relation to the health state of the individual (Fig 3a). These community-wide profiles display unique communities in each individual, corresponding to the results in Fig 2. For example, while the microbial communities of participants A, B, and C are dominated by *Pseudomonas*, participants D, E, and F have more diverse communities consisting of dominant organisms such as *Prevotella*, *Streptococcus*, and *Fusobacterium* (Fig 3a). These communities, generated using 16S rRNA gene sequencing, mirror the selective culturing performed by the clinical microbiology laboratory associated with the clinic (Table 1); however, greater diversity is evident via 16S rRNA gene sequencing approaches.

During the study period, all participants except for C experienced a pulmonary exacerbation (Fig 3a, red triangles). Unfortunately, no samples were obtained from participant A during a pulmonary exacerbation that occurred between samples A8 and A9. Visually, we observe from these taxa summaries that there are sometimes, but not always, observable changes in the lung microbiota preceding, during, or following pulmonary exacerbations.

To quantify these observations, the Bray-Curtis dissimilarity between samples (Fig 3b) and statistical measurements between categories were calculated (S2 Table). These metrics indicate that there are statistically significant changes in the lung microbiota between non-Treatment (Intermediate and Stable) and Treatment time points in 2 of the 4 participants (S2 Table; participants A and C were omitted due to no Treatment samples). These community-wide changes are seen between participant B’s Intermediate and Treatment time points (p = 0.045) as well as in participant E (Stable vs. Treatment, p = 0.022; Intermediate vs. Treatment, p = 0.009). However, results from participants D and F indicate no statistically significant changes to the microbiome with Treatment (S2 Table). Importantly, in participant E who had 4 exacerbations in the study period, only 1 of the 4 was accompanied with statistical changes to the microbiota (S2 Table). None of these observed alterations in the microbiota were accompanied with statistically significant changes to FEV1 (S3 Fig, S3 Table).

Further, there are observable disturbances to the lung microbiota within treatment categories. Fig 3b indicates changes in the lung community between a number of sequentially collected samples, taken at least 1 month outside of any exacerbation. Examples include changes in Bray-Curtis dissimilarity scores between Samples A1 and A2 as well was Samples B2 and B18 when compared to other Stable time points. Together, these findings indicate that some but not all exacerbations (2 of 7) result in or are preceded by a discrete, measurable change in the microbiome and that observable shifts in these communities also occur independent of exacerbation onset.

### Exacerbation is not linked with changes in within-sample diversity

**Fig 4:**
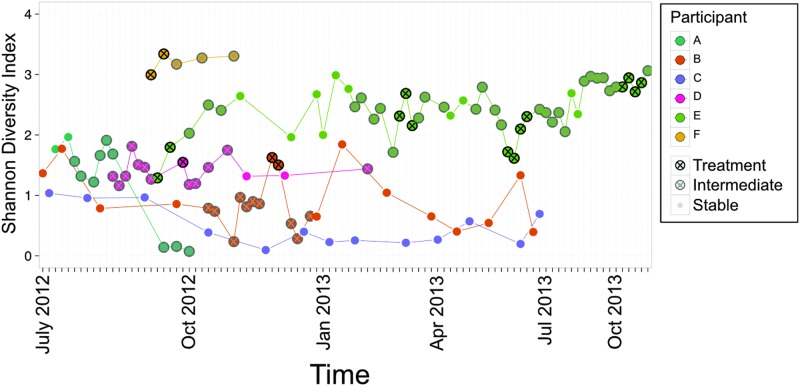
Diversity within the lung community does not consistently decrease with exacerbation. A longitudinal representation of the evenness and richness of the CF lung microbiota across study participants indicates patient-specific levels of within-patient diversity.

Each sample within this study was examined independently to determine the within sample diversity by calculating Shannon’s diversity index, an alpha diversity metric measuring both richness and evenness. Previous research has reported a decrease in alpha diversity with declining lung function and age [38], [39], affected by antibiotic therapy [40] and exacerbation treatment [41], [42]; increased diversity of the lung microbiota is associated with stable lung function [43]. In this study, differences in Shannon’s diversity were measured between Treatment, Intermediate, and Stable samples in each individual (Fig 4, S4 Fig). While a significant increase in diversity was observed between Intermediate and Treatment samples in Participant B (S4b Fig), the majority of samples showed no significant differences between Treatment, Intermediate, and Stable samples (Fig 4, S4 Fig).

**Fig 5:**
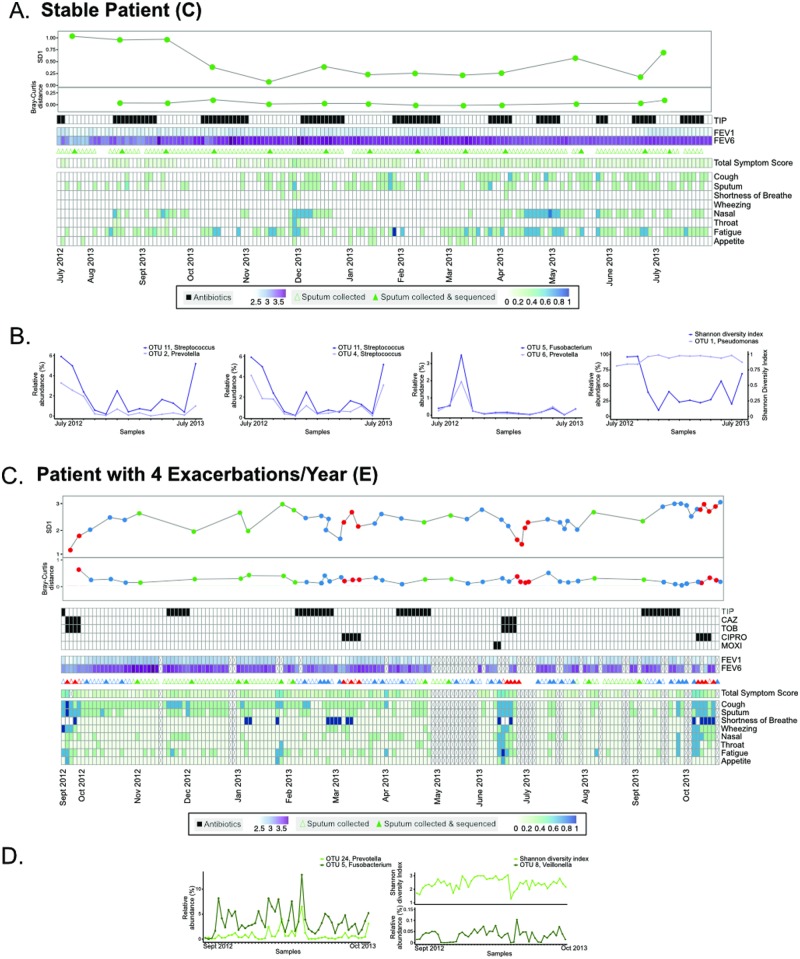
Longitudinal dynamics of two select participants (C and E). Two participants who were the outliers in terms of the number of pulmonary exacerbations experienced over the course of the study period were chosen for closer examination. **A**. Sample collection for participant C is shown in relation to, antibiotic use, FEV1, and symptom scores. **B**. Correlations between collected data, diversity metrics, and OTU relative abundance were calculated and significant correlations were reported (S4 Table); a subset of these significant correlations are plotted. **C**. Sample collection for participant E in relation to antibiotic use, FEV1, and symptom scores. **D**. Correlations between these collected data and the OTUs present within the microbiome were calculated and significant correlations were reported (S5 Table); a subset of these significant correlations are plotted.

**Fig 6:**
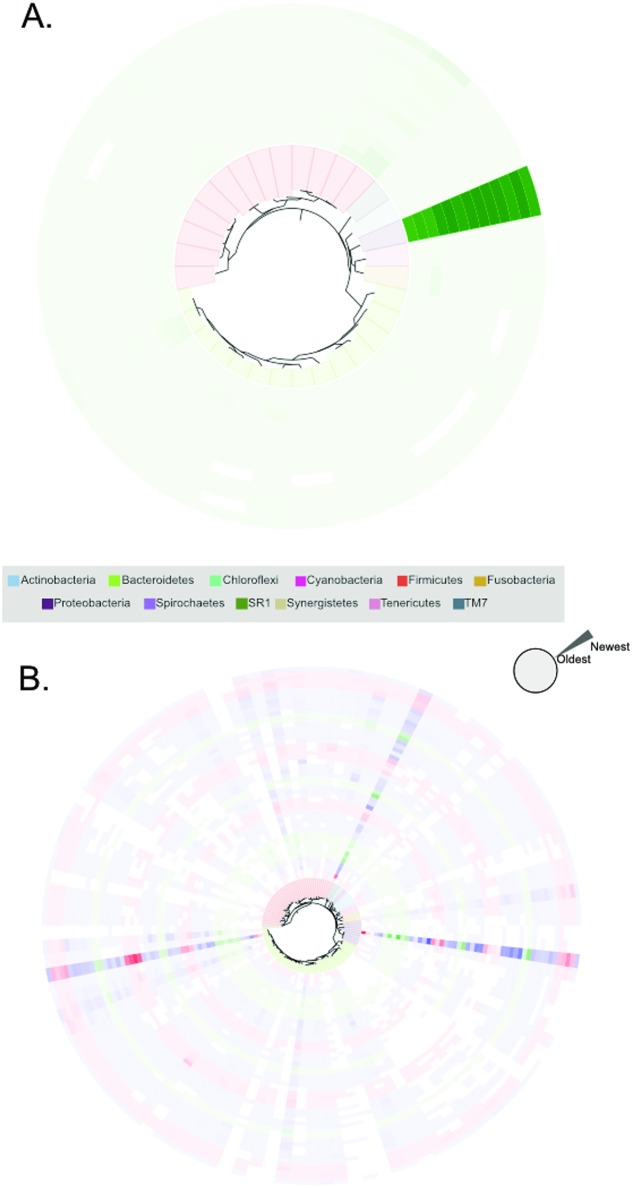
Examples of stability and variability in the CF lung microbial communities of two select participants (C and E). **A**. Visualization of the stability of participant C's lung microbial community over the study period. Each OTU is presented as a terminal node on the phylogeny; its presence in each sample evaluated using 16S rRNA gene sequencing is shown extending outwardly from the inner phylogeny in chronological order. The density of the color indicates the relative abundance of the OTU; when the OTU is not identified, the space is left blank. **B**. Participant E, who experienced 4 exacerbations over the course of the year, has a much more variable lung microbiota than participant C. Similar to Fig 5c, OTUs are represented as nodes in the phylogeny whose relative abundance is indicated with varying color density. Rings in the phylogeny are colored to indicate the sample type (Treatment red, Intermediate blue, Stable green). Density of the color indicates relative abundance of the OTU and time periods are colored according to the health state.

Furthermore, Shannon’s diversity index was patient-specific (Fig 4). A range of values (0.077–3.345) were observed across participants (Fig 4). Interestingly, the participant with the lowest mean diversity score was the only participant who did not experience an exacerbation during the study period (Fig 4, participant C, purple line) and who maintained the highest FEV1 over the course of the study (Table 1, Figs 5 and 6). Although this sample size is small, these findings indicate that the use of alpha diversity metrics to assess the relative health status and stability of the lung microbiota may be complex at the patient-level even though low alpha diversity has been associated with poor lung function at the population-level.

### Longitudinal dynamics of the CF lung microbiota

To further understand elements of the patient-specific dynamics of the CF lung microbiota, we focused on 2 participants who completed the full study period. We chose participants C and E because they represented the individuals who had the least (n = 0, C) and most (n = 4, E) observed exacerbations.

As has been shown above, participant C demonstrated fairly uniform alpha and beta diversity across the study period (Fig 3b and Fig 4). This individual was on alternating 4-week tobramycin inhalation powder (TIP) therapy throughout the year, and had a consistent FEV1 in the range of 2.2–2.69L (Fig 5a). Overall, this individual's symptom scores were low (i.e. close to baseline), although there were periods of increased sinus congestion and fatigue during the study period. Correlations were calculated using eLSA between all core OTUs (sum > 1.0% relative abundance across all samples from participant), collected data (antibiotic use, FEV1, symptom scores), and diversity metrics (Shannon diversity index, Bray-Curtis dissimilarity scores) (Fig 5b, S4 Table). None of the collected data correlated with individual components of the microbiota; of the diversity metrics tested, Shannon diversity was negatively correlated with OTU 1 (Fig 5b). Instead, correlating OTUs within the microbiome were observed (Fig 5b, S4 Table). For example, *Prevotella* OTU 2 was positively correlated with *Streptococcus* OTU 11 (Fig 5b); additionally, OTU 11 was correlated with another *Streptococcus* (OTU 4). Further, *Prevotella* (OTU 8) was positively correlated with *Fusobacterium* OTU 5. However, all of these correlations were observed amongst OTUs with low (<10%) relative abundance. When the relative abundance of each OTU was examined longitudinally, we observed a remarkably stable lung microbial community dominated by a single *Pseudomonas* OTU (Fig 6a). These results suggest a community within the lung whose composition is highly dependent on its microbial membership, but less on external factors such as antibiotic use.

However, when we examine participant E, we see a very different picture of CF lung disease. Participant E was also on alternating TIP therapy over the study period; however, this treatment was supplemented with further antibiotics upon exacerbation onset including ceftazidime (CAZ), tobramycin (TOB), ciprofloxacin (CIPRO), and moxifloxacin (MOXI) (Fig 5c). FEV1 decreased over the study period and was within a range of 1.13–2.11L. Similar to participant C, when correlations between OTUs, collected data, and diversity metrics were calculated, we found no correlations between OTUs and collected data such as antibiotic use, FEV1, and symptom scores (S5 Table). *Fusobacterium* OTU 5 was positively correlated with *Prevotella* OTU 24 over the study period (Fig 5d). Additionally, as observed with participant C, Shannon diversity was correlated with multiple OTUs (Fig 5d, S5 Table). Participant E's lung community was consistently dominated by 3 OTUs corresponding to *Pseudomonas*, *Prevotella*, and *Streptococcus* (Fig 6b). In contrast to the stable microbiome seen in participant C, the community in participant E contained many members which fluctuated over the study period (Fig 6b).

## Discussion

Our current understanding of the pathophysiology of pulmonary exacerbations in CF is limited. Understanding the mechanisms underlying pulmonary exacerbation, and thus being able to mitigate symptom onset and/or severity would have important implications for individuals with CF. Pulmonary exacerbations likely have many triggers including elements of the inflammatory response, lung microbiota, and extrinsic factors such as pollution, allergen exposure and medication compliance [5]. Because antimicrobial therapies often control and resolve the symptoms associated with pulmonary exacerbations, it is important that we understand the longitudinal dynamics of the CF lung microbiota with respect to onset of pulmonary symptoms.

In this study, when examined as discrete groups, samples of the CF lung microbiota obtained during Treatment, Intermediate, and Stable periods were identified as significantly different from each other (PERMANOVA of Bray-Curtis distance, p = 0.016) though highly confounded by the originating participant. However, when samples from each participant were examined independently, it was evident that discrete changes in microbial composition only accompanied some pulmonary exacerbations (Fig 3). Further, longitudinal analyses did not provide statistically significant correlations between respiratory symptoms and elements of the lung microbiota (Figs 5 and 6). Notably, although changes are seen during some participant's pulmonary exacerbations, some individual's lung microbiota also undergo large compositional changes during periods of clinical stability. These types of changes may result from changes in antimicrobial therapy [17]; [21], changes in pulmonary function [20]; [44], or other undetermined factors.

When we focused on the 2 participants in the study who had the most (n = 4) and least (n = 0) number of exacerbations during the study period, longitudinal analyses were unable to provide general microbiome patterns predicting exacerbation; there were no correlations between exacerbation and alpha or beta diversity, FEV1, antibiotic use, or symptom scores. Previous longitudinal analyses of the CF lung microbiota's role in pulmonary exacerbation onset have drawn similar conclusions to those observed within this study [22]; [23]. Instead, statistically significant correlations between alpha diversity and microbial membership (i.e. OTUs) were identified, as well as correlating OTUs. In both participants there was a negative correlation found between dominating members of the microbiota and alpha diversity. However, these correlations are likely a result of the compositional and relative nature of the 16S rRNA gene sequencing approaches employed. In participant C, positive correlations were observed between *Prevotella* and *Streptococcus*, as well as between 2 *Streptococci*. In both participants, correlations were observed between *Prevotella* and *Fusobacterium*. These species are often found in the lungs of individuals with CF, but haven't been previously correlated. However, *Streptococcus salivarius* and *Prevotella intermedia* have been implicated in coaggregation in periodontal disease [45], and oral streptococci and *Prevotella* have been isolated together from dentoalveolar abscesses [46], indicating that organisms within these genera may correlate in a variety of infectious diseases. While these correlations did not differ before, during, or after pulmonary events, they may be important microbe-microbe interactions in this environment which should be further investigated. It is important to note that because of imperfections in OTU clustering approaches [47]; [48], that the 2 correlating streptococci OTUs may in fact be sequences from the same organism which were misclustered into 2 separate OTUs.

In this study, we identified inter-individual differences of the CF lung microbiota in terms of taxonomic composition (Fig 3), alpha (Fig 4), and beta diversity (Fig 2). The results of this study help us to consider the goal of this research: to better understand and improve the lives of those suffering from CF. Studying 6 participants longitudinally has identified that conclusions which have been made in the literature which apply at the population-level are not necessarily meaningful to the individual. For example, we report that periods of exacerbation were not consistently correlated with an increase in Shannon Diversity (Fig 4). This is in contrast to previous results which have shown increases in alpha diversity during exacerbations when compared to surrounding time points at the population-level [41]; [42]. It has been previously suggested that a patient-specific, cross-sectional use of alpha diversity to predict state of disease would not be of use [20], especially since measures of alpha diversity cannot be acted on in the clinic via a specified treatment or pharmacological aid. Because of the unique nature of microbial acquisition in the lungs, CFTR modulators, and patient environments and actions, individuals with CF represent unique patients who should be assessed in a case-by-case basis.

The most important limitation of this study are the short-comings of using 16S rRNA sequencing of sputum as a measure of the CF lung microbiome. First, 16S rRNA sequencing does not distinguish nonviable from viable cells. Second, the onset of exacerbations may be triggered by a small proportion of the total community or by non-bacterial members of the microbiota; however, this method does not differentiate between metabolically active and inactive members and does not capture non-bacterial components [19]. Third, although conflicting studies exist [49], expectorated sputum may be subject to contamination by oral microbes [39][50]. These important shortcomings of our ability to fully understand the CF lung microbiome may mean that with the advantages that 16S rRNA sequencing of sputum affords (total community profiling with relative abundance information), that the associated disadvantages may be masking an important microbial component to these events.

A small sample size of participants enrolled and completed the study (Table 1). Prospectively collecting and storing sputum samples is tedious and difficult in a large patient cohort. Previous studies were similarly limited to small patient numbers [22]; [23]. Additionally, requesting tri-weekly symptom score profiles and self-administered spirometry measurements furthers the participant burden on individuals with a disease that already requires time-consuming pharmacologic and physical therapies [51]. However, longitudinal studies of the dynamics within the CF lung microbiota are important in determining the bacterial component of pulmonary exacerbation.

By studying 6 people with CF for up to a year in a prospective, longitudinal study of the microbiota preceding, during, and following exacerbation, we conclude no discernable, participant-wide dynamics which explain the onset of pulmonary exacerbation. Some hypothesized causes of pulmonary exacerbations may not have been measurable in this study; for example, we have previously hypothesized that strain dynamics would be very difficult to determine from community-wide studies of the 16S rRNA gene [19]. Further, elements other than the microbiome, such as host inflammatory defenses, may be the driving force behind these events. This study also supports the growing data that suggest that the lung microbiome in CF is highly patient-specific and that it should be investigated as such.

## Supporting information

S1 Fig Flowchart of the 16S rRNA gene sequencing data processing approach. Paired-end 16S rRNA gene sequencing data was processed using custom perl scripts which tied together existing processing software. These software, including their versions, options used, and order are presented here for the purposes of reproducibility.
(TIFF)

S2 Fig Genus-level biplot of the Participant-dependent CF lung microbiome. A biplot of PC1 vs. PC2 of the PCoA plot displayed in Fig 2 reveals specific genera which contribute to Participant-specific separation. Taxonomic label text is scaled to represent the mean relative abundance of each genera across the dataset. PC = Principal Coordinate.
(TIFF)

S3 Fig Longitudinal FEV1 values for each participant over the study period. FEV1 data were collected 3x a week over the study period. Red vertical bars indicate Treatment time points.
(TIFF)

S4 Fig Alpha diversity measures of each participant over the study period. Shannon diversity index was calculated for each microbiota sample collected over the study period. Statistical analyses between sample types indicated a significant difference between Intermediate and Stable samples from Participant A and Treatment and Intermediate time points in Participant B. All other comparisons were not statistically significant.
(TIFF)

S1 Table Study duration and sample information. (DOCX)

S2 Table p-values of statistical comparisons of Bray-Curtis dissimilarity scores between groups. (DOCX)

S3 Table p-values of statistical comparisons of FEV1 changes between groups. (DOCX)

S4 Table Significantly correlating OTUs and select metadata for Particpant C. LS = local similarity score; PCC = pearson coorelation coefficient.
(DOCX)

S5 Table Significantly correlating OTUs and select metadata for Participant E. LS = local similarity score; PCC = pearson coorelation coefficient.
(DOCX)
