# Supplementary figures and images for: Longitudinal sampling of the lung microbiota in individuals with cystic fibrosis
Source: PLoS One. 2017 Mar 2;12(3):e0172811. doi: 10.1371/journal.pone.0172811 (PMC5333848; doi:10.1371/journal.pone.0172811)

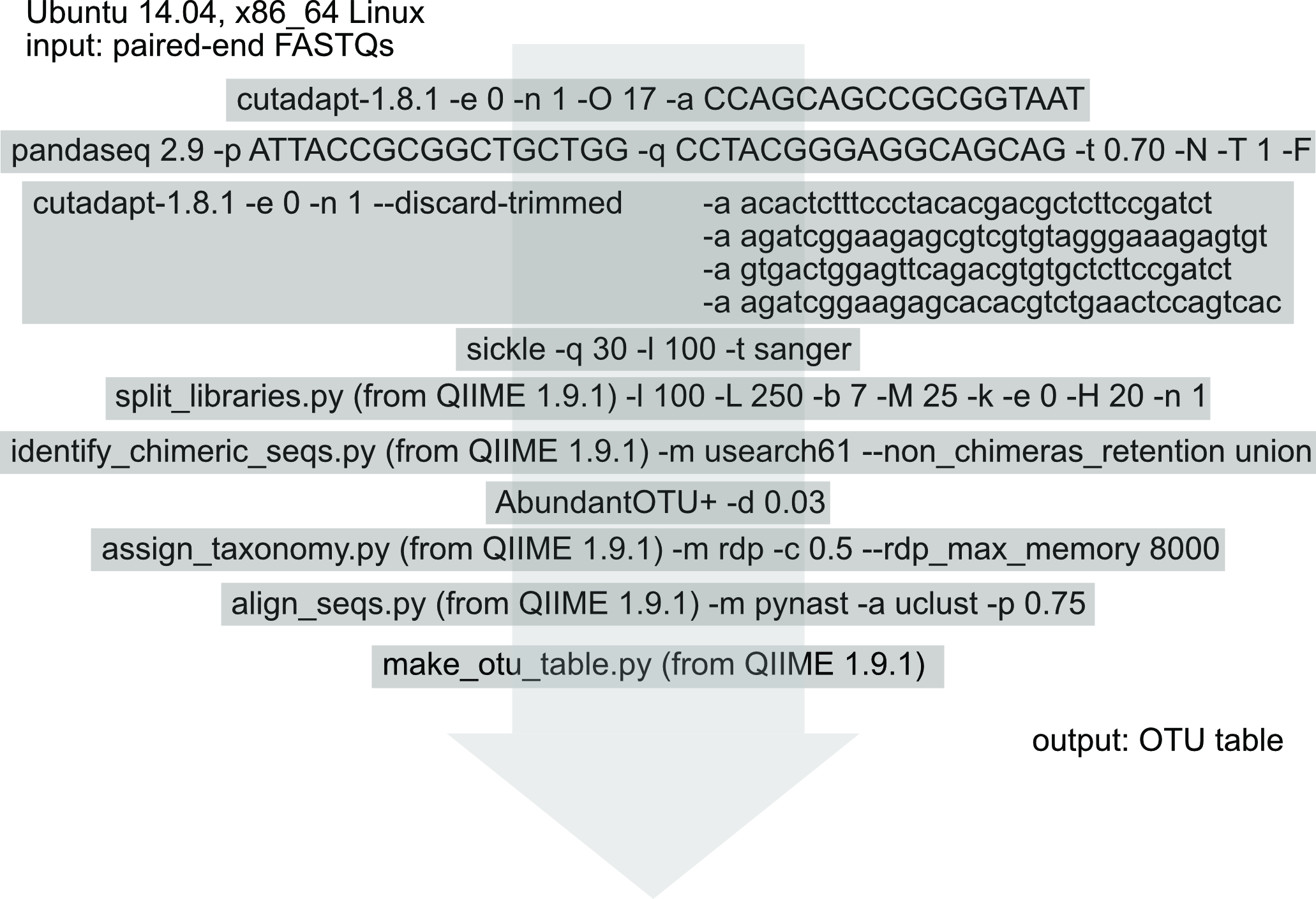

Supplement: S1 Fig — Paired-end 16S rRNA gene sequencing data was processed using custom perl scripts which tied together existing processing software. These software, including their versions, options used, and order are presented here for the purposes of reproducibility. (TIFF) [file pone.0172811.s001.tiff]

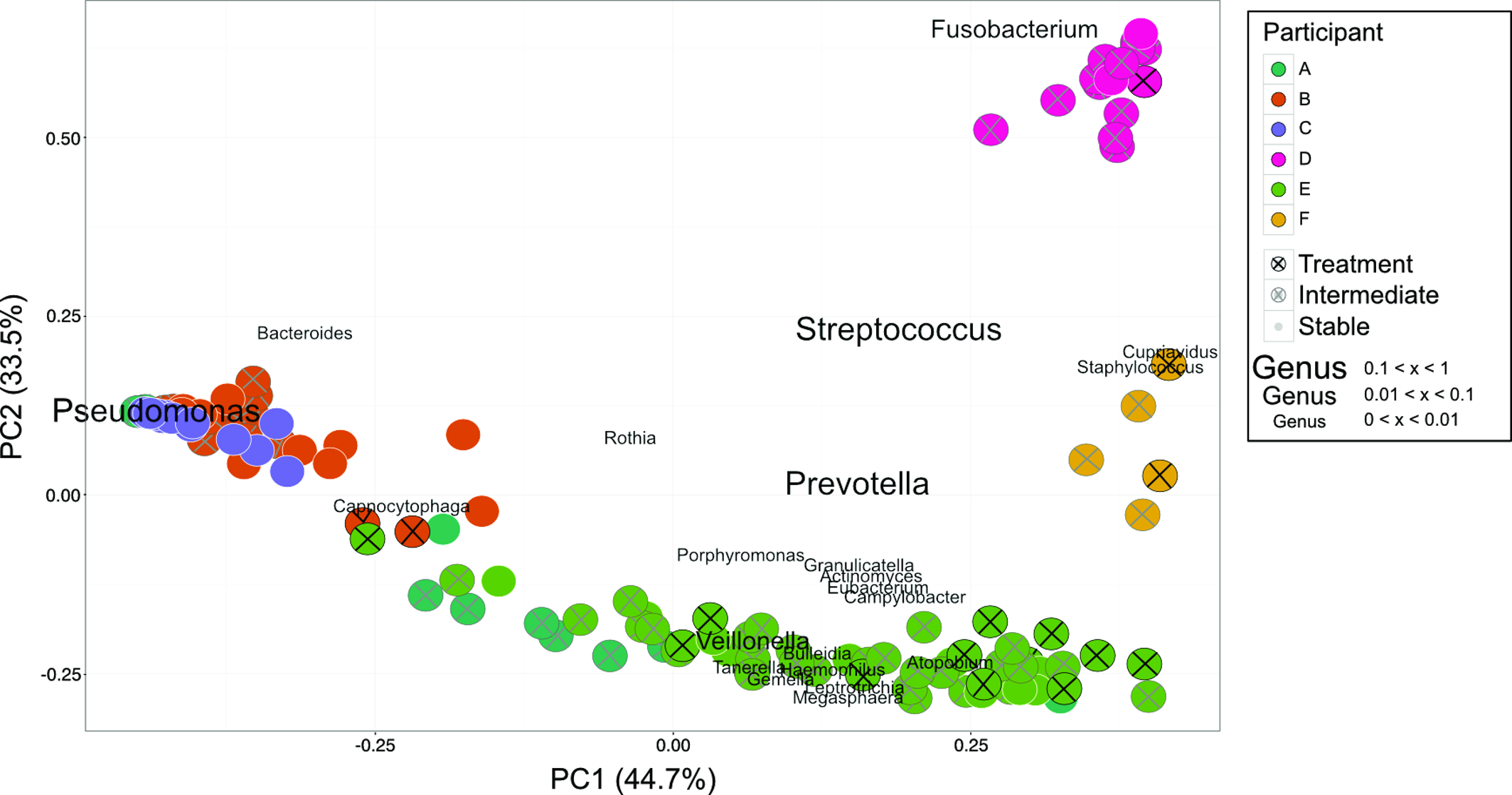

Supplement: S2 Fig — A biplot of PC1 vs. PC2 of the PCoA plot displayed in Fig 2 reveals specific genera which contribute to Participant-specific separation. Taxonomic label text is scaled to represent the mean relative abundance of each genera across the dataset. PC = Principal Coordinate. (TIFF) [file pone.0172811.s002.tiff]

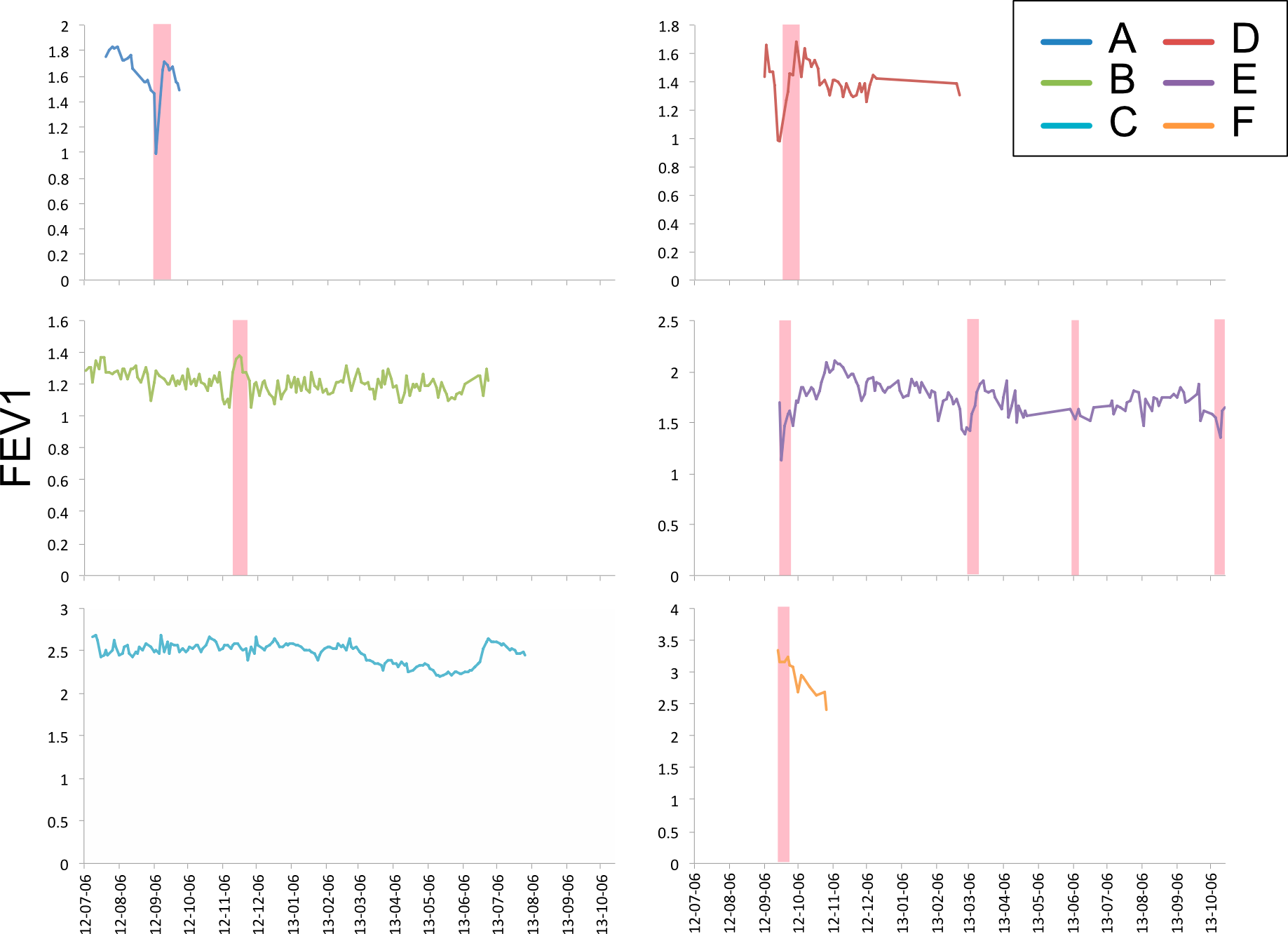

Supplement: S3 Fig — FEV1 data were collected 3x a week over the study period. Red vertical bars indicate Treatment time points. (TIFF) [file pone.0172811.s003.tiff]

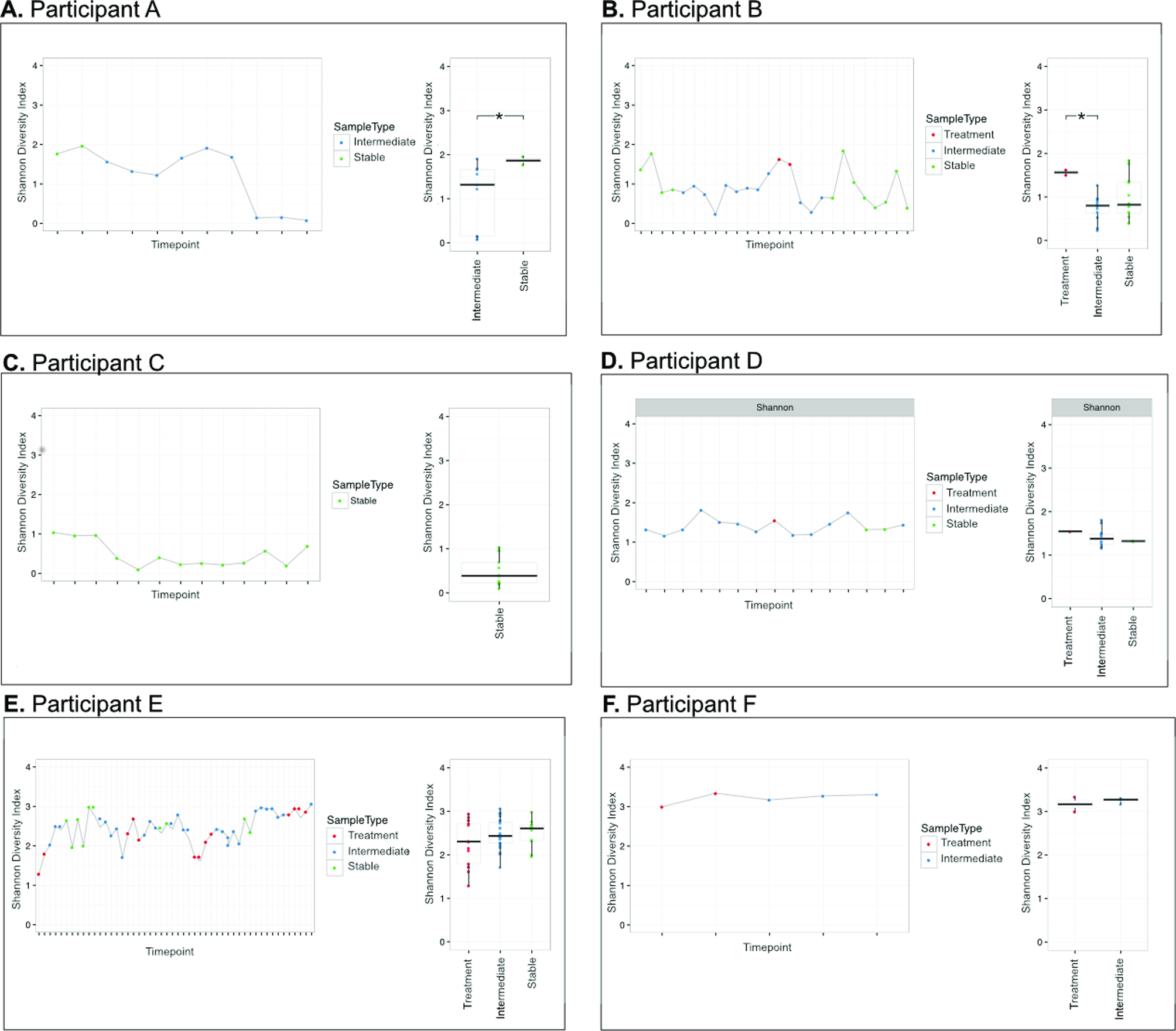

Supplement: S4 Fig — Shannon diversity index was calculated for each microbiota sample collected over the study period. Statistical analyses between sample types indicated a significant difference between Intermediate and Stable samples from Participant A and Treatment and Intermediate time points in Participant B. All other comparisons were not statistically significant. (TIFF) [file pone.0172811.s004.tiff]
