# Supplementary material for: Longitudinal sampling of the lung microbiota in individuals with cystic fibrosis
Source: PLoS One. 2017 Mar 2;12(3):e0172811. doi: 10.1371/journal.pone.0172811 (PMC5333848; doi:10.1371/journal.pone.0172811)
Supplement: S1 Table — (DOCX) [file pone.0172811.s005.docx]

**Table S1. Study duration and sample information.**

| **Participant** | **Study Duration** | | **# of Collected Samples** | **# of Sequenced Samples** |
| --- | --- | --- | --- | --- |
|  | **Dates** | **Length (in days)** |  |  |
| A* | 2012 | 66 | 21 | 11 |
| B | 2012-2013 | 360 | 143 | 26 |
| C | 2012-2013 | 384 | 152 | 13 |
| D* | 2012-2013 | 236 | 26 | 15 |
| E | 2012-2013 | 398 | 154 | 51 |
| F* | 2012 | 45 | 12 | 5 |

* did not complete study
