# Supplementary material for: Longitudinal sampling of the lung microbiota in individuals with cystic fibrosis
Source: PLoS One. 2017 Mar 2;12(3):e0172811. doi: 10.1371/journal.pone.0172811 (PMC5333848; doi:10.1371/journal.pone.0172811)
Supplement: S2 Table — (DOCX) [file pone.0172811.s006.docx]

**Table S2. p-values of statistical comparisons of Bray-Curtis dissimilarity scores between groups.**

| **Participant** | **Stable vs. Intermediate** | **Stable vs. Treatment** | **Intermediate vs. Treatment** |
| --- | --- | --- | --- |
|  |  |  |  |
| A | 0.137 | - | - |
| B | 0.662 | 0.17 | **0.045** |
| C | - | - | - |
| D | 0.451 | 0.333 | 0.218 |
| E | 0.765 | **0.022** | **0.009** |
| E1 | 0.8 | 0.5 | 0.4 |
| E2 | 0.76 | 0.832 | 0.601 |
| E3 | 0.643 | 0.067 | **0.002** |
| E4 | 0.745 | 0.4 | 0.499 |
| F | - | - | 0.9 |
