# Supplementary material for: Longitudinal sampling of the lung microbiota in individuals with cystic fibrosis
Source: PLoS One. 2017 Mar 2;12(3):e0172811. doi: 10.1371/journal.pone.0172811 (PMC5333848; doi:10.1371/journal.pone.0172811)
Supplement: S3 Table — (DOCX) [file pone.0172811.s007.docx]

**Table S3. p-values of statistical comparisons of FEV1 changes between groups.**

| **Participant** | **Stable vs. Intermediate** | **Stable vs. Treatment** | **Intermediate vs. Treatment** |
| --- | --- | --- | --- |
|  |  |  |  |
| A | **0.036** | - | - |
| B | **0.035** | 0.67 | 0.102 |
| C | - | - | - |
| D | 0.806 | - | - |
| E | 0.089 | 0.009 | 0.067 |
| E1 | **0.029** | 0.232 | 0.304 |
| E2 | 0.334 | 0.633 | 0.925 |
| E3 | - | - | 0.436 |
| E4 | - | - | 0.289 |
| F | - | - | 0.183 |
