# Supplementary material for: Longitudinal sampling of the lung microbiota in individuals with cystic fibrosis
Source: PLoS One. 2017 Mar 2;12(3):e0172811. doi: 10.1371/journal.pone.0172811 (PMC5333848; doi:10.1371/journal.pone.0172811)
Supplement: S4 Table — LS = local similarity score; PCC = pearson coorelation coefficient. (DOCX) [file pone.0172811.s008.docx]

**Table S4. Significantly correlating OTUs and select metadata for Participant C.**

| **X** | **Y** | **LS** | **PCC** | **Length** | **p-value** | **q-value** |
| --- | --- | --- | --- | --- | --- | --- |
| OTU1;g_Pseudomonas | OTU2;g_Prevotella | -0.887973 | -0.950445 | 13 | 0.005497 | 0.043444 |
| OTU1;g_Pseudomonas | OTU4;g_Streptococcus | -0.920999 | -0.897723 | 13 | 0.003601 | 0.043444 |
| OTU1;g_Pseudomonas | OTU8;g_Veillonella | -0.906857 | -0.913167 | 13 | 0.004302 | 0.043444 |
| OTU1;g_Pseudomonas | OTU9;g_Prevotella | -0.873737 | -0.867662 | 13 | 0.006531 | 0.043444 |
| OTU1;g_Pseudomonas | OTU11;g_Streptococcus | -0.935891 | -0.907245 | 13 | 0.003007 | 0.043444 |
| OTU1;g_Pseudomonas | Alpha | -0.99053 | -0.981703 | 13 | 0.001428 | 0.043444 |
| OTU2;g_Prevotella | OTU8;g_Veillonella | 0.941744 | 0.941562 | 13 | 0.002695 | 0.043444 |
| OTU2;g_Prevotella | OTU9;g_Prevotella | 0.95917 | 0.937007 | 13 | 0.002161 | 0.043444 |
| OTU2;g_Prevotella | OTU11;g_Streptococcus | 0.873311 | 0.863102 | 13 | 0.006531 | 0.043444 |
| OTU2;g_Prevotella | OTU12;g_Prevotella | 0.951426 | 0.927755 | 13 | 0.002414 | 0.043444 |
| OTU2;g_Prevotella | Alpha | 0.909055 | 0.928795 | 13 | 0.004152 | 0.043444 |
| OTU4;g_Streptococcus | OTU11;g_Streptococcus | 0.945851 | 0.936433 | 13 | 0.002599 | 0.043444 |
| OTU4;g_Streptococcus | Alpha | 0.892802 | 0.840943 | 13 | 0.005128 | 0.043444 |
| OTU5;g_Fusobacterium | OTU6;g_Prevotella | 0.973557 | 0.978646 | 13 | 0.001792 | 0.043444 |
| OTU5;g_Fusobacterium | OTU28;g_Prevotella | 0.967562 | 0.995455 | 13 | 0.001932 | 0.043444 |
| OTU5;g_Fusobacterium | Alpha | 0.857732 | 0.578402 | 13 | 0.008006 | 0.047114 |
| OTU6;g_Prevotella | OTU28;g_Prevotella | 0.961842 | 0.984822 | 13 | 0.002082 | 0.043444 |
| OTU8;g_Veillonella | OTU9;g_Prevotella | 0.903566 | 0.932725 | 13 | 0.004456 | 0.043444 |
| OTU8;g_Veillonella | OTU11;g_Streptococcus | 0.872449 | 0.883309 | 13 | 0.006531 | 0.043444 |
| OTU8;g_Veillonella | OTU12;g_Prevotella | 0.886775 | 0.951942 | 13 | 0.005497 | 0.043444 |
| OTU8;g_Veillonella | Alpha | 0.929193 | 0.914985 | 13 | 0.003232 | 0.043444 |
| OTU9;g_Prevotella | OTU11;g_Streptococcus | 0.901649 | 0.896837 | 13 | 0.004616 | 0.043444 |
| OTU9;g_Prevotella | OTU12;g_Prevotella | 0.880315 | 0.933035 | 13 | 0.006098 | 0.043444 |
| OTU9;g_Prevotella | Alpha | 0.875753 | 0.841578 | 13 | 0.006311 | 0.043444 |
| OTU11;g_Streptococcus | Alpha | 0.918932 | 0.845682 | 13 | 0.003732 | 0.043444 |
| OTU12;g_Prevotella | Alpha | 0.872279 | 0.80161 | 13 | 0.006531 | 0.043444 |

LS = local similarity score; PCC = Pearson correlation coefficient
