# Supplementary material for: Longitudinal sampling of the lung microbiota in individuals with cystic fibrosis
Source: PLoS One. 2017 Mar 2;12(3):e0172811. doi: 10.1371/journal.pone.0172811 (PMC5333848; doi:10.1371/journal.pone.0172811)
Supplement: S5 Table — LS = local similarity score; PCC = pearson coorelation coefficient. (DOCX) [file pone.0172811.s009.docx]

**Table S5. Significantly correlating OTUs and select metadata for Participant E.**

| **X** | **Y** | **LS** | **PCC** | **Length** | **p-value** | **q-value** |
| --- | --- | --- | --- | --- | --- | --- |
| OTU1;g_Pseudomonas | OTU13;g_Prevotella | -0.533465 | -0.497165 | 50 | 0.000653 | 0.01071 |
| OTU4;g_Streptococcus | OTU7;g_Streptococcus | 0.48208 | 0.408235 | 50 | 0.002599 | 0.027483 |
| OTU4;g_Streptococcus | OTU67;g_Rothia | 0.691045 | 0.666126 | 50 | 0.000004 | 0.00035 |
| OTU4;g_Streptococcus | OTU103;g_Streptococcus | 0.799579 | 0.795728 | 50 | 0 | 0.000017 |
| OTU5;g_Fusobacterium | OTU24;g_Prevotella | 0.534316 | 0.621561 | 50 | 0.000627 | 0.01071 |
| OTU7;g_Streptococcus | Alpha | 0.575569 | 0.477824 | 50 | 0.000188 | 0.004889 |
| OTU8;g_Veillonella | Alpha | 0.521608 | 0.499919 | 50 | 0.000897 | 0.012957 |
| OTU9;g_Prevotella | Alpha | 0.446739 | 0.353607 | 50 | 0.006311 | 0.047559 |
| OTU13;g_Prevotella | OTU25;g_Bulleidia | 0.559686 | 0.560181 | 50 | 0.0003 | 0.006502 |
| OTU19;g_Prevotella | OTU74;f_Streptococcaceae | -0.459854 | -0.245866 | 50 | 0.004616 | 0.041437 |
| OTU23;g_Prevotella | OTU33;g_Prevotella | 0.561707 | 0.583158 | 50 | 0.000287 | 0.006502 |
| OTU25;g_Bulleidia | OTU55;g_Peptostreptococcus | 0.480291 | 0.228019 | 50 | 0.002695 | 0.027483 |
| OTU27;g_Prevotella | Alpha | 0.672771 | 0.532487 | 50 | 0.000008 | 0.00047 |
| OTU74;f_Streptococcaceae | OTU101;g_Azorhizophilus | 0.887692 | 0.900804 | 50 | 0 | 0.000001 |
| OTU74;f_Streptococcaceae | OTU103;g_Streptococcus | 0.485105 | 0.331119 | 50 | 0.002414 | 0.026155 |

LS = local similarity score; PCC = Pearson correlation coefficient
